# Psychosis following a stroke to the cerebellum and midbrain: a case report

**DOI:** 10.1186/s40673-015-0037-8

**Published:** 2015-12-09

**Authors:** Michael Bielawski, Helen Bondurant

**Affiliations:** Department of Psychiatry, University of Alberta, 1E1 Walter Mackenzie Health Science Centre (WMC), 8440 112 St NW, Edmonton, AB T6G 2B7 Canada; Royal Alexandra Hospital, 10240 Kingsway Avenue NW, Edmonton, AB T5H 3V9 Canada

**Keywords:** Post-stroke psychosis, Cerebellar cognitive affective syndrome, Cerebellar stroke, Cerebellum, Psychosis

## Abstract

**Background:**

There is growing evidence that the cerebellum serves an important role in controlling affect and cognition, and its pathology has been implicated in several psychiatric disorders. Furthermore, the brainstem’s role in cognition and affect has been historically overlooked. Neuroimaging studies and an increasing number of case reports indicate cognitive deficits and hallucinatory phenomena after isolated brainstem lesions.

**Case presentation:**

We report a 56-year-old man who developed persistent persecutory delusions, hallucinations, cognitive impairment and flattened affect following an extensive bilateral cerebellar stroke with involvement of the midbrain.

**Conclusions:**

This is one of the few reported cases of unremitting psychosis secondary to cerebellar and mesencephalic vascular infarction. We suggest, based on the distribution of the patient’s lesions, that his corresponding symptoms are a result of a disruption to cerebrocerebellar pathways. This article briefly reviews recent pathophysiological explanations behind the psychosis associated with brainstem and cerebellar lesions, the treatment, as well as the relation of these structures to each other.

## Background

The cerebellum has a well-established role in modulating motor control and its dysfunction manifests as disorders of ataxia, dysdiadochokinesia, dysmetria, dysarthria, diplopia, and dysphagia [1, 2]. In more recent times, the cerebellum has become recognized as having a wider encompassing involvement in modulating higher order cognitive and emotional processing [3]. Experimental and clinical research on patients with cerebellar pathology has identified a pattern of dysfunction in the domains of executive function (e.g., impaired working memory, decreased verbal fluency, poor planning), spatial cognition (e.g., disrupted visual spatial organization and memory), affect (e.g., flat affect or inappropriate behavior) and language (e.g., dysprosodia and agrammatism) [2]. Schmahmann coined the changes as a “cerebellar cognitive affective syndrome (CCAS)” [2]. CCAS is also referred to by its eponym Schmahmann’s Syndrome [4]. Pathology of the cerebellum has been linked to various psychiatric disorders, which is attributed to the disruption of the circuitry connecting the cerebellum to other cerebral structures including limbic structures [3]. For example, schizophrenia has alternatively been described, pathophysiologically, as a “cognitive dysmetria” due to dysfunction in the cortico-cerebello-thalamo-cortical circuit [5]. We report a case of psychosis involving persecutory delusions, auditory and visual hallucinations secondary to a vascular infarct affecting bilateral cerebellar hemispheres and part of the midbrain. Few published cases have described infratentorial stroke lesions producing a persisting psychosis. We will briefly discuss contemporary theories about the pathophysiology of psychosis induced by cerebellar-brainstem lesions.

## Case presentation

This is a case of a 56-year-old male with an unremarkable past psychiatric and family history. He was previously healthy except for having untreated hypertension, dyslipidemia, and being a tobacco chewer. He was married with children, and was a semi-retired business owner.

**Fig. 1:**
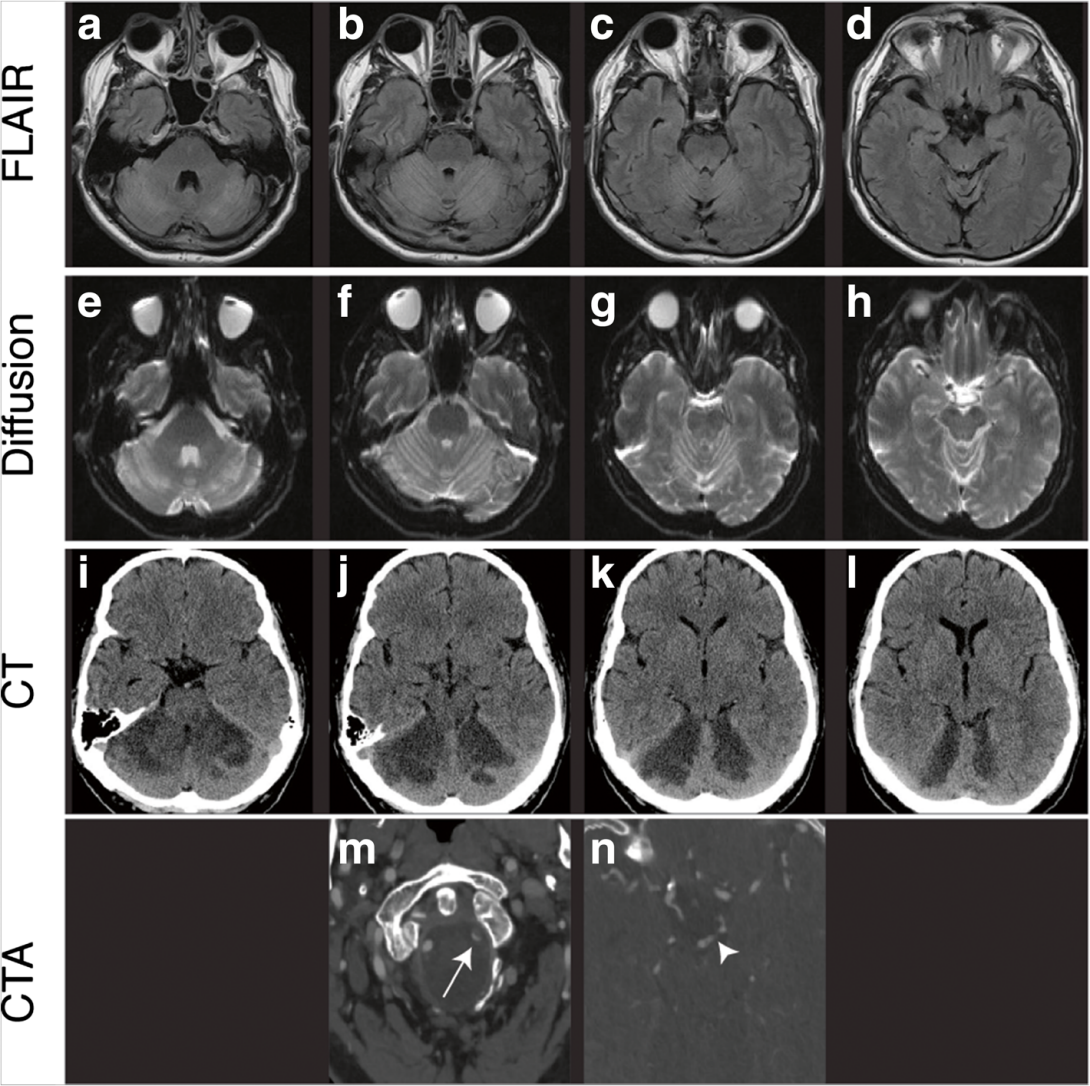
Axial 1.5 T MR, CT and CTA images demonstrate bilateral SCA infarct and left vertebral artery dissection. **a**–**d** Sequential axial FLAIR MR images demonstrate bilateral cerebellar hemisphere hyperintensity superiorly with relative sparing inferiorly (not shown) and involvement of the colliculi in the midbrain and the superior cerebellar peduncles. **e**–**h** Sequential axial diffusion MR images demonstrate restricted diffusion in both cerebellar hemispheres in the same distribution of the FLAIR images. **i**–**l** Sequential axial non-contrast CT images demonstrate patchy areas of hypoattenuation bilaterally in the superior aspects of the cerebellar hemispheres. **m**–**n** Select axial CTA images demonstrate decreased caliber of left vertebral artery with arterial lumen filling defect (arrow). As well a filling defect is present in the basilar terminus extending to the P1 segment of the left PCA (arrowhead). *MR* magnetic resonance, *FLAIR* fluid-attenuated inversion recovery, *CT* computer tomography, *CTA* computer tomography angiography, *SCA* superior cerebellar artery, *PCA* posterior cerebral artery

His initial presentation to the emergency occurred after a sudden onset of severe headache, dizziness, nausea, vomiting, blurry vision, slurred speech, and ataxia. His initial CT scan was inconclusive but a subsequent MRI later the same day demonstrated extensive acute bilateral cerebellar infarcts with some involvement of the midbrain colliculi and superior cerebellar peduncles (Fig. 1). A CT angiogram of the head and neck performed on the same day indicated an intracranial left vertebral artery dissection with embolus to the distal basilar artery extending into the P1 segment of the left posterior cerebral artery. Bilateral SCA territory infarcts were indicated by patchy areas of hypoattenuation within the bilateral cerebellar hemispheres as well as the inferior right and bilateral superior cerebellar lobes (Fig. 1).

The patient had otherwise unremarkable findings from medical investigations including a normal transthoracic echocardiogram. During his admission to the stroke neurology unit, the patient suffered from ongoing significant ataxia, dysarthria, scanning speech, motor dysmetria, and mild diplopia. He required a temporary nasogastric tube feed and subsequent insertion of a peg tube for 3 months due to dysphagia. There was mild right-sided hemisensory impairment to sharp touch and temperature, which resolved after 4 months. His other neurological symptoms gradually improved but did not completely resolve over his nearly 5-month admission to hospital. Dysmetria and ataxia were most resistant to rehabilitation.

Approximately 2 days into his admission, records indicated that the patient began experiencing hallucinations and was thought to be “confused.” The patient described seeing cameras and police in his room at night. He expressed delusions such as “Russians are coming to get me”, “police were stealing people away at night”, and became suspicious that his wife was committing adultery. The patient’s perceptual disturbances were more pronounced at night. Extensive laboratory investigations were performed along with repeat head CT scans and a review of his medications -- all results were non-indicative for delirium. After 3 weeks, his symptoms persisted despite maintaining orientation in all three spheres. The consulting psychiatrist diagnosed the patient with “psychotic disorder due to stroke” based on the observation that the patient suffered from delusions and hallucinations with a lack of insight. He was started on 2.5 mg Olanzapine at night. Consequently, the patient reportedly slept more at night although accounts of his paranoid delusions and agitation persisted. The anxiety associated with his perceptual disturbances appeared to diminish over the next 3 weeks until discharge.

The patient was transferred to an inpatient stroke rehabilitation hospital for another 3 months and Olanzapine was discontinued on admission as per his request. Psychiatry was re-consulted as the patient expressed ongoing fears of being harmed by intruders in the hospital and complained of hearing people being shot at night, which affected his sleep. He believed that SWAT members were consistently lurking in the hallways. He lacked insight about his fixed ideas and endorsed his beliefs throughout the day to his wife and staff. His affect was flat and minimally reactive during assessments. His speech was slow and monotone but he could express himself coherently otherwise. Other than anxiety related to his delusions the patient denied experiencing problems with depression. A repeat brain MRI disclosed no new findings. The patient was diagnosed with a “persistent post-stroke psychosis”. He agreed to restarting Olanzapine and the dose was gradually increased to 5 mg in the evenings. Same as before, the patient’s delusions and hallucinations did not subside but he reported significantly less anxiety about his delusions and had better sleep.

On the cognitive level, based on formal tests involving the Brixton Spatial Anticipation Test, Indendent Living Scale, Repeatable Battery for the Assessment of Neuropsychological Status, and Weschler Memory Scale (WMS-III: Spatial Span Subtest), the patient was impaired in the following domains: divided attention/multitasking (mild impairment), visual-spatial working memory (mild impairment), and immediate and delayed memory for verbal information (mild to moderate impairment). Novel problem-solving ability was in the low average range. Family members and health care staff commented that the patient showed impulsivity in behavior resulting in unsafe transfers and having a “shorter fuse.” In contrast, the patient was found to have above average language and fine visual-spatial judgment of angles and distances. Auditory working memory fell within normal limits.

The patient was scheduled for a follow-up appointment with outpatient psychiatry in one month’s time but he did not attend. He was subsequently lost to follow-up.

## Discussion

The patient in this case lacked any pre-morbid psychiatric history or family history of mental illness. The onset of psychotic symptoms shortly after vascular insult strongly suggested a post-stroke psychosis caused by lesions appearing on diagnostic imaging. The distribution of our patient’s lesions overlapped the anterior and posterior cerebellar lobes. Damage to the lateral regions of each hemisphere was most pronounced with relatively mild involvement of the vermis. Specific lobules affected were II–VI in the left hemisphere and II–VII in the right hemisphere. Schmahmann outlined the cerebellum’s topography of function; linking lobules I–V to sensorimotor function, and lobules VI, VII to cognitive and affective function [1]. Our patient’s significant motor ataxia could be explained by damage to lobules II–V. His blunted affect, impulsivity, executive deficits and delusions may be related to injured lobules VI and VII, which is consistent with the CCAS [1]. Studies by Schmahmann had previously shown that affective disturbances were routinely occurring in patients with damage to the vermis [4] and cognitive function was affected by lateral parts of the posterior lobe [1]. Furthermore, cognitive and affective changes were more prominent in bilateral hemisphere pathology [4].

Given the increasingly established evidence for the cerebellum’s role in higher order processing, it is understandable that in this case and in others [6–11], damage to the cerebellum or to its cortical pathways can result in affective and cognitive disturbances manifesting as a psychotic disorder. The previously proposed concept of “cognitive affective psychosis” [11] may be applicable in this case. The pathophysiological mechanism subserving the development of psychosis is uncertain but it can be understood in the context of a cognitive dysmetria as postulated in patients with schizophrenia [5]. The cerebellum, through its network of Purkinje cells and deep nuclei, modulates incoming information through pattern and error detection [5]. The dysfunctional cerebellum will misinterpret an internal signal from auditory cortex as an external stimulus leading to flawed output back to cerebral cortex resulting in an auditory hallucination experience [5]. The cerebellum participates in associated learning [5]. When this function is disrupted, perceptions become connected with an “erroneous matrix of associations” leading to misinterpreted events experienced as delusions [5].

Damage to the patient’s midbrain may have further precipitated the patient’s symptoms due to its potential involvement in the cerebrocerebellar network. Through SPECT studies and review of cases in the literature, Marien and D’aes [12] demonstrated the brainstem to be an essential part of the cerebrocerebellar network, and its interruption of excitatory input to the cerebrum and cerebellum can result in a pattern of deficits similar to the CCAS. It is suggested that the brainstem can modulate cognition and affect through reciprocal connections with the cerebellum and the cerebrum [13].

## Treatment

Our patient had partial response to Olanzapine on both 2.5 and 5 mg evening doses. Mainly sleep and anxiety were improved but abatement of psychotic symptoms was questionable. The dose may have been inadequate or perhaps Olanzapine’s receptor profile was inadequate for the region of pathology. To the best of our knowledge, there is no consensus on preferred pharmacological treatment of psychotic symptoms secondary to cerebellar lesions. There are limited case reports on treatment, however one case reported success with a combination of Clozapine and Valproate [10]. Another article reported success in treating a patient’s depressive and psychotic symptoms with Fluoxetine and Risperidone [11].

## Conclusion

This case highlights the importance of both the cerebellum and the brainstem in influencing behavior, cognition and affect, functions that historically have been overlooked in these structures. There is mounting evidence in the literature that lesions to the brain in the distribution of the posterior circulation are capable of producing sophisticated disturbances in behavior and perception. Increased awareness of this phenomenon will hopefully result in greater recognition and understanding of the psychiatric and cognitive deficits of infratentorial lesions.

## Consent

Written informed consent was obtained from the patient for the publication of this report and any accompanying images.

## References

[1] Schmahmann JD (2004). Disorders of the cerebellum: ataxia, dysmetria of thought, and the cerebellar cognitive affective syndrome. J Neuropsychiatry Clin Neurosci.

[2] Schmahmann JD, Sherman JC (1998). The cerebellar cognitive affective syndrome. Brain.

[3] Shakiba A (2014). The role of the cerebellum in neurobiology of psychiatric disorders. Neurol Clin.

[4] Manto M, Mariën P (2015). Schmahmann’s syndrome - identification of the third cornerstone of clinical ataxiology. Cerebellum Ataxias.

[5] Andreasen NC, Pierson R (2008). The role of the cerebellum in schizophrenia. Biol Psychiatry.

[6] Emul M (2010). Co-occurrence of psychiatric symptoms with cerebellar venous malformation: a case report. J Neuropsychiatry Clin Neurosci.

[7] Miyazawa T, Ito M, Yasumoto Y (2009). Peduncular hallucinosis following microvascular decompression for trigeminal neuralgia without direct brainstem injury: case report. Acta Neurochir (Wien).

[8] Lu ML, Yeh IJ (2001). Onset of psychosis after cerebellum pathology: a case report. Gen Hosp Psychiatry.

[9] Jurjus GJ, Weiss KM, Jaskiw GE (1994). Schizophrenia-like psychosis and cerebellar degeneration. Schizophr Res.

[10] Almeida J (2011). Effective treatment with clozapine and valproate for refractory schizophrenia-like psychosis after cerebellar hemorrhage. Clin Neuropharmacol.

[11] Duggal HS (2005). Cognitive affective psychosis syndrome in a patient with sporadic olivopontocerebellar atrophy. J Neuropsychiatry Clin Neurosci.

[12] Marien P, D’Aes T (2015). “Brainstem cognitive affective syndrome” following disruption of the cerebrocerebellar network. Cerebellum.

[13] D’Aes T, Marien P (2015). Cognitive and affective disturbances following focal brainstem lesions: a review and report of three cases. Cerebellum.

